# A Great Sanitarian

**Published:** 1904-07-30

**Authors:** 


### The Hospital.

A Journal of

The Medical Sciences and Hospital Administration.

Vol. XXXVI.—No. 931. JULY 30, 1904.

### A Great Sanitarian.

The death of Sir John Simon, K.C.B., which occurred on Saturday, the 23rd instant, in his eighty-eighth year, removes from the medical profession one of the most notable figures of the past century. An eminent surgeon or physician does not, as a rule, exert any wide or far-reaching control over public health or general mortality, and the exceptions to this rule may be counted upon the fingers. Sir Spencer Wells, among those who have been taken away, Lord Lister, among those who remain, are certainly the most notable, even if they be not the sole examples; and of these it may be conceded that Lord Lister alone can really challenge any comparison with the great founder of the science of public sanitation. Simon was already surgeon to St. Thomas's Hospital, and had already, by the publication of his lectures on pathology, given evidence of a remarkable power of grasping and of applying principles, when, in 1848, he accepted the position of medical officer of health to the City of London. In that capacity his reports in successive years were models alike of scientific boldness and of scientific caution; dealing with such subjects as house-drainage, water-supply, the social condition of the poor, houses unfit for habitation, offensive and injurious trades, and interments; but it was in the report for 1853, when, after four years of absence, Asiatic cholera had again shown itself in London, that he struck the keynote of his future work, and laid down the law that in given densities of population, the prevalence of the disease would mainly vary with the degree in which that dense population lived in the atmosphere of its own excrements and refuse. The principle thus stated was found every year to be of wider and wider application; and, in his farewell address to the City, printed in 1854 as a preface to the collected series of his reports, Simon pointed out the "wild absurdity of seeking at Peru what here runs to waste beneath our pavements, of ripening only epidemic disease with what might augment the food of the people, of waiting, like our ancestors, to expiate the neglected divinity of water in some bitter purgation by fire." He added, with almost prophetic insight, that it needed a grasp of political mastership, not uninformed by science, to convert obvious elements of knowledge to practical application, to recognise a national object irrelevant to the interests of party, to lift an universal requirement from the sphere of professional jealousies, and to found in immutable principles the sanitary legislation of a people.

Mr. Simon's transference from the service of the City of London to that of the Government in the successive capacities of Medical Officer to the General Board of Health, to the Privy Council, and to the Local Government Board, had the necessary effect of at once widening his own outlook and of enabling him to speak with increased authority and to larger audiences. The work which was commenced in 1858 by his Introductory Report to the General Board of Health on the Sanitary State of the people of England, a report in which he declared that, of the entire annual mortality of the country, "at least a fourth part is of artificial production," was continued year after year with constantly increasing diligence and with constantly increasing effect. His position enabled him to guide and direct the inquiries of the Government medical inspectors, and also to obtain assistance from skilled workers outside the office; and the results of their labours, harmonised and systematised by the master, were annually given to the world in the official reports which soon formed a priceless body of sanitary literature, and were in demand in every civilised country. In this way Netten Radcliffe was enabled to obtain absolute proof of the conveyance of cholera by drinking water contaminated by choleraic excrement; and Buchanan to demonstrate the diminution of illness and mortality, and especially of phthisis mortality, which, in 16 great towns, had been the immediate consequence of improvements in soil drainage, in excrement disposal, and in water supply. Every accumulation of evidence enabled Simon to fasten his eminently practical mind more and more securely upon the leading idea of "filth" as the chief factor in the causation of disease, and the reforms which he and his inspectors never ceased to urge upon the authorities of suffering localities have undoubtedly been the chief causes of that constant diminution of the death-rate which for so many years has been in progress, and which does not even now appear to have reached its limit. In the five years from 1838 to 1842 London had an average mortality of 25 per thousand; and in 1902, among a population at least trebled in number, the death-rate was 17 per thousand. The saving which these figures express was, in the main, the work of Sir John Simon.

The Public Health Act of 1872, the passing of which Simon's labours had greatly tended to promote, led indirectly to the close of his official career. The Government of the day was largely dependent upon the votes of the owners of nuisances and of insanitary property, and the administration of the Act was arranged with a single view of avoiding offence to them. The lives of the people were as nothing in the estimation of professional politicians, whose hold upon power was mainly dependent upon the success of their appeals to prejudice and ignorance, and Simon, after a period of struggle with his official superiors, resigned his office. He was appointed a Crown Member of the General Medical Council, and in that capacity was mainly instrumental in securing that degrees and diplomas in public health should be given under such precautions as really to represent proficiency. The high level of the public health service is almost entirely due to his strenuous exertions, and does not form the smallest of his claims upon the gratitude of the nation which he served so well.

